# A model for representing the semantics of MWEs: From lexical semantics to the semantic annotation of complex predicates

**DOI:** 10.3389/frai.2023.802218

**Published:** 2023-03-23

**Authors:** Voula Giouli

**Affiliations:** ATHENA Research Centre, Institute for Language and Speech Processing, Maroussi, Greece

**Keywords:** verbal MWEs, semantic representation, lexical semantics, linguistic ontology, semantic relations, Semantic Role Labeling (SRL)

## Abstract

Multiword expressions (MWEs) are sequences of words that pose a challenge to the computational processing of human languages due to their idiosyncrasies and the mismatch between their phrasal structure and their semantics. These idiosyncrasies are of lexical, morphosyntactic and semantic 11 nature, namely: non-compositionality, i.e., the meaning of the expression cannot be computed from the meanings of its constituents; discontinuity, i.e., alien elements may intervene; non-13 substitutability, i.e., at least one of the expression constituents is lexicalized and therefore, does not enter in alternations at the paradigmatic axis; and non-modifiability, in that they enter in syntactically 15 rigid structures, posing further constraints over modification, transformations, etc. The paper presents a model for representing MWEs at the level of semantics by taking into account all these inherent idiosyncrasies. The model assumes the form of a linguistic ontology and is applied to Greek verbal multi-word expressions (VMWEs); moreover, the semantics of the lexical entries under scrutiny is also represented via the semantics of their arguments based on corpus evidence. In this regard, modeling the semantics of VMWEs is placed in the lexicon-corpus interface.

## 1. Introduction

MWEs are highly idiosyncratic structures (Gross, 1982, 1998a,b; Lamiroy, 2003; Baldwin and Kim, 2010; Constant et al., 2017) and thus considered “a pain in the neck for Natural Language Processing” (Sag et al., 2002). In terms of meaning, they appear in a continuum of compositionality, which ranges from expressions that are very analysable to others that are partially analysable or ultimately non-analysable at all (Nunberg et al., 1994). However, most MWE-specific lexical resources focus on the representation of their properties at the levels of morphology and syntax only overlooking their semantic representation; similarly, although several datasets (corpora, lexica, tools) have been developed in view of training and evaluating algorithms for MWE identification and discovery, relatively little work has been devoted to the semantics of MWEs.

Our work seeks to fill this gap by proposing a model for encoding the semantic properties of VMWEs into a lexical resource by considering all the idiosyncrasies they exhibit. The semantics of VMWEs are thus defined along the following axes: (a) the type of VMWE in terms of the degree of compositionality, (b) their mapping onto concepts or word senses already existing in an inventory, that is, a semantic lexical resource already available; (c) at the paradigmatic axis, *via* encoding the lexical semantic relations between a VMWE and other single- or multi-word entries; and (d) at the syntagmatic axis, by modeling the semantics of their arguments based on corpus evidence. In the latter case, the VMWE is taken as a whole, that is, as a complex predicate. Our goal is to treat both single- and multi-word entries in a comparable way that would be useful for Natural Language Processing (NLP) applications.

## 2. Related work

### 2.1. Modeling MWEs in lexical resources

Most Lexical Resources (LRs) dedicated to MWEs give an account only of their lexical, morphological, and syntactic idiosyncrasies. Within the Lexicon-Grammar framework, the pioneering work of Gross (1982) toward the analysis and classification of French VMWEs resulted in the formal representation of their syntactic and distributional properties, selectional restrictions and in the signaling of their fixed as opposed to non-fixed constituents in the so-called Lexicon-Grammar tables; along the same lines, similar LRs based on the same formal principles and linguistic criteria have been created for idiomatic expressions in other languages, as for example Greek (Fotopoulou, 1993; Mini, 2009). Similarly, Villavicencio (2004) notice that providing a uniform lexical encoding for all types of MWEs is a difficult task to undertake due to their idiosyncratic nature, proposing, thus, a set of requirements for the efficient representation of English idioms and verb-particle constructions (VPCs) in lexica by means of augmenting existing single- word dictionaries with specific tables. Similarly, MWE-specific lexicons provide elaborate linguistic information for subcategorization, internal modification, etc. (Grégoire, 2010; Zaninello and Nissim, 2010; Shudo et al., 2011; Odijk, 2013); yet they do not account for their semantic representation. Even lexical resources that provide recommendations for representing MWEs in mono- and multilingual computational lexica (Calzolari et al., 2002; Copestake et al., 2002) focus mainly on the syntactic and semantic properties of support verbs and noun compounds and their proper encoding thereof.

However, the quest for representing word meanings in NLP lexicons has been for decades the focus of attention in NLP, often taking linguistic theories of lexical semantics into account. In this respect, SIMPLE semantic lexica (Busa et al., 2001), intended for 12 European languages (Catalan, Danish, Dutch, English, Finnish, French, German, Greek, Italian, Portuguese, Spanish, and Swedish) were developed as harmonized lexica around an upper level ontology and on top of pre-existing morphological and syntactic lexica; based on the Generative Lexicon theory (Pustejovsky, 1995) and the notion of *Qualia Structure*, SIMPLE lexica encode structured semantic types and semantic (subcategorization) frames. A few years later, the Brandeis Semantic Ontology (BSO) seeks to extend the English SIMPLE lexicon (Pustejovsky et al., 2006). At the syntax-semantics interface, SynSemClass (Urešová et al., 2018a,b), is a bilingual synonym lexicon organized on the basis of contextually based synonymy and valency of verbs in a bilingual setting; at the heart of the bilingual lexicon lays the analysis of semantic “equivalence” (synonymy or near synonymy) of verb senses, and their valency behavior in parallel Czech-English language resources. In this respect, semantic MWE-aware lexicons, i.e., WordNet (Fellbaum, 1998), Verbnet (Kipper et al., 2008), SAID (Kuiper et al., 2003), and WikiMwe (Hartmann et al., 2012) give an account of various types of MWEs—yet they are solely focused on their semantic representation overlooking other aspects. Similarly, MWEs in FrameNet (Baker et al., 1998) are represented from the perspective of their semantic heads, the latter being concerned with the mapping of meaning to form *via* the theory of Frame Semantics (Fillmore, 1968). Along the same lines, the mapping of MWEs onto concepts is proposed in Fotopoulou et al. (2014), Hawwari et al. (2014) and Fotopoulou and Giouli (2017).

### 2.2. Modeling MWEs in corpora

Besides lexical resources, corpus annotation projects also seek to model MWEs. In this regard, a comprehensive – yet shallow – annotation of heterogeneous mwes in running text is presented in Schneider et al. (2014); similarly, the DiMSUM 2016 shared task for joint identification and supersense tagging of nominal and verbal MWEs (Schneider et al., 2016) developed training and test data in English (tweets, service reviews, and TED talk transcriptions). Similarly, within the PARSEME initiative, corpora in more than 20 languages were developed in view of discovery and identification of VMWEs (Savary et al., 2017; Ramisch et al., 2018, 2020); annotation is performed based on annotation guidelines which are as universal as possible, but which still allow for language specific categories and tests. More recently, a dataset in English, Portuguese and Galician was developed within the SemEval-2022 Task 2 on multilingual idiomaticity detection; the task was aimed at identifying whether a sentence contains an idiomatic expression, and at representing potentially idiomatic expressions in context based on semantic text similarity.

Other MWE-aware corpora include treebanks (Abeillé et al., 2003; Vincze et al., 2010; Bejček et al., 2012) also coupled with sense annotations (Adesam et al., 2015) or corpora devoted to Semantic Role Labeling (SRL), that is, the task of assigning semantic roles as defined in Dowty (1991) and Van Valin (1993, 1999) to the arguments of predicates.Viewed as a level of shallow semantic analysis aimed at representing events and their participants, the task is considered as an intermediate level of semantic representation that can help map from syntactic parse structures to deeper, more fully specified representations of meaning. In this respect, SRL has been proved to improve Natural Language Tasks, as for example, Question-Answering (Shen and Lapata, 2007), Machine Translation (Shi et al., 2016), Information Extraction (Bastianelli et al., 2013).

In this context, the Proposition Bank (PropBank) is one of the earliest corpora annotated with semantic roles (Palmer et al., 2005). In PropBank, role definitions are determined for each verb depending on its meaning; semantic roles in PropBank are verb-sense specific. Besides verbs, noun, and adjective predicates as well as Light Verb Constructions (LVCs) and Idiomatic Expressions (IEs) are assigned one or more semantic role(s) depending on their meaning (Bonial et al., 2014a,b). Light Verb Constructions in PropBank are treated in two consecutive passes: at the first pass, the light verb is annotated as appropriate by selecting (or creating) the relevant. LV roleset; annotation proper is performed on the predicative noun at the second pass. In all cases, one of the main drawbacks of this schema is that Arg2-Arg5 are not consistent, causing, thus, inconsistencies in labeling.

Contrary to PropBank in which roles are specific to a verb, semantic roles in FrameNet (Baker et al., 1998) are specific to a frame. In this context, semantic roles assume the form of frame elements. For each frame, a set of core semantic roles (called core frame elements) are generally assumed as central to the meaning conveyed by the frame. The resulting frame annotation scheme is therefore rather fine-grained. One step further, the Abstract Meaning Representation (AMR) corpus provides construction-based annotations for a variety of semi- and non-compositional phrases considering PropBank lexicon and framesets (Bonial et al., 2018).

The inconsistencies attested in PropBank due to the under-specificity of semantic roles have been addressed in VerbAtlas (Di Fabio et al., 2019), a large-scale, handcrafted semantic lexical resource aimed at bringing together all verbal synsets from WordNet into semantically-coherent frames. Indeed, one of the major contributions of VerbAtlas is the definition of cross-domain explicit semantic roles.

## 3. A model for representing the semantics of VMWEs

Taking as a starting point the Saussurian notion of the *linguistic sign*, the model we propose builds on the principles of Semantic field theory and assumes the form of a linguistic ontology (Fotopoulou and Giouli, 2017; Giouli and Sidiropoulos, 2020), with two building blocks (main classes), namely, the SIGNIFIER and the SIGNIFIED. The ontology builds on the model proposed by Markantonatou et al. (2010) with significant extensions and modifications as documented in Fotopoulou and Giouli (2017). Each entry in the ontology is encoded as a unique combination of a form (a word form), instantiated under the SIGNIFIER class and a concept; the latter is an instance of the class SIGNIFIED.

The encoding of MWEs with rich linguistic information revealing their morphological idiosyncrasies, combinatorial preferences (surface structure), and syntactic properties at the SIGNIFIER level has been extensively presented in Fotopoulou et al. (2014). According to the specifications, MWEs are initially assigned a grammatical category based on their function as Noun, Verb, Adjective, or Adverb. Next, MWEs are further labeled with respect to the degree of fixedness (Sag et al., 2002) as *fixed, semi*-*fixed*, and *syntactically flexible*. Their surface structure is further specified, along with information about their fixed elements as opposed to non-fixed ones. In our lexicon model, each MWE structure is represented as a Part-of-Speech sequence following the Lexicon-Grammar notation. VMWEs in specific, are labeled based on the classification proposed in Fotopoulou (1993) and Mini (2009). According to the respective notation, *N* denotes a non-fixed nominal, whereas C signifies a fixed one; numbers are used to represent the syntactic function of fixed or nonfixed constituents. In this sense, *N0* is used to represent a non-fixed argument in subject position whereas, C0 denotes a fixed subject. Similarly, *N1, N2, N3*, etc., along with *C1, C2, C3* etc. denote complements in object position (or complements of prepositional phrases), marked also for fixedness. Possible syntactic properties (i.e., subcategorization information, syntactic alternations, etc) are also encoded at this level. In the next sections, we elaborate on the encoding at the level of semantics. Our model provides mechanisms for encoding diathesis alternations, register, and for signaling MWEs that have a literal (and compositional meaning) besides their idiomatic one, as defined in Savary et al. (2019).

The semantic representation of lexical items—both single- and multi-word ones—is achieved at the SIGNIFIED level, taking into account the following aspects: (a) coarse classification that reflects their degree of compositionality; (b) mapping onto word senses or concepts; (c) linking with other entries *via* semantic relations, and (d) identifying their arguments and the roles they assume. We will elaborate on the model itself in the next paragraphs.

### 3.1. Typology of VMWEs: Degree of compositionality

VMWEs are assigned a label reflecting their degree of compositionality based on the typology and specifications proposed within the PARSEME Shared Task initiative (Savary et al., 2017; Ramisch et al., 2018, 2020), it is compatible with 1.2 annotation guidelines^1^, and makes extensive use of the decision flowcharts provided therein; based on linguistic tests and criteria, these decision trees allow for the consistent classification of candidate VMWEs. Greek VMWEs fall in the following categories: (a) *verbal idiomatic expressions* (VIDs), that bear a meaning that cannot be computed based on the meaning of their constituents and the rules used to combine them; (b) *light verb constructions* (LVCs), i.e., expressions with a rather transparent meaning; (c) *multi-verb constructions* (MVCs), that is, expressions with coordinated lexicalised head verbs [i.e., απ*o*ρώ και εξíσταμαι (= to question-myself and be-very-surprised, to be very surprised)]; and (d) *verb-particle constructions* (VPCs) comprising a verb and one of the adverbs μπρ*o*στ ά (=in front), πíσω (=back), π άνω (up), κ άτω (=down), μ έσα (=in), έξω (=out, outside) in Greek; these adverbs are not morphologically derived from adjectives and exhibit most - if not all - of the properties particles in other languages have Giouli et al. (2019)^2^. Given their resemblance with VPCs in other languages, we decided to retain the latter class for Greek, and therefore expressions as the ones depicted in (1) and (2) were classified as VPCs. In terms of their semantics, VPCs were identified to have a non-compositional meaning. Note however, that they are the most ambiguous ones since, depending on the context, they can also be used literally bearing a fully compositional meaning—in which case they are not VMWEs (Savary et al., 2019).

(1) π έϕτω μ έσα
  *lit*. fall_1−sg_ in (=to succeed in a prediction, to predict correctly)
(2) π έϕτω έξω
  *lit*. fall_1−sg_ out (=to get bankrupt)

In terms of meaning, the classification in the afore-mentioned classes is a first step toward defining their semantics: VIDs and MVCs are non-compositional, LVCs are semi-compositional, in that they have a transparent meaning which is retained by the predicative noun, whereas VPCs present semantic ambiguity. Of course, other dimensions exist along which these different types of VMWEs can also be compared, namely, non-modifiability, and non-substitutability. In this regard, VIDs, VPCs and MVCs are syntactically rigid structures posing constraints with respect to modification, syntactic transformations, or other alternations at the paradigmatic axis etc., as opposed to the more flexible LVCs.

### 3.2. Conceptual representation of VMWEs

**Figure 1:**
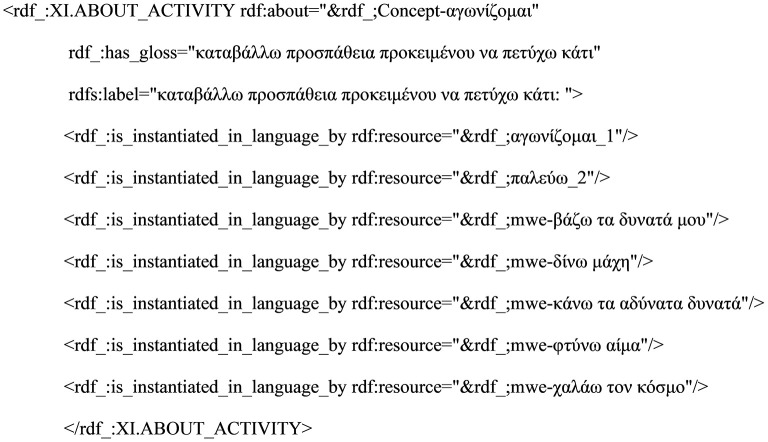
Lexical entries under the concept αγωνζoμαι.

At the next level, the semantic representation of VMWEs makes use of the SIGNIFIED branch of our ontology, and each VMWE (like all other MWEs and single words) is mapped onto a concept. In our model lexicon, concepts are treated as instances under hierarchically organized (sub-)classes; these sub-classes are themselves subsumed under a set of top-level classes (or top ontology) and roughly correspond to the notion of semantic or lexical fields (Lyons, 1977, p. 268). In this respect, concepts are grouped together in terms of some relatedness or closeness of meaning, and in a way, they are conceived of as homogenous sets of synonymous or near-synonymous words. Classes are further populated with one -or more- word forms from the SIGNIFIER class, as shown in Figure 1. Following common lexicographic practices, a gloss provided for each concept guides the inclusion of entries under the concept.

Lexical entries (words) are then linked together *via* lexical semantic relations: synonymy, near-synonymy, antonymy; similarly, concepts are also linked together *via* semantic relations as appropriate: hypernymy-hyponymy or is_a relation, meronymy, etc. Apart from the standard lexical semantic relations, other relations are also included: entailment (*entails*), causation (*causes*), temporal order (*happens_before)*, etc. Moreover, relations that link together words and/or concepts that belong to different grammatical categories have been defined in the resource. For example, relations of the type *is_the_agent_of*, *feels_emotion, is_cogniser*, etc. link together concepts instantiated by verbs denoting an activity, an emotion or a cognitive state and concepts instantiated by nouns denoting the actor, the experiences of the cognitive agent, etc. Similarly, relations that link together adjectives with adverbs have been used. In total, more than 100 relations have been employed so far; some of them are generic in that they are relative to more than one semantic field (as, for example the relations *Is_a, Is_part_of, Is_member_of, Consists_of, Is_Agent_of*, etc), whereas others are domain-specific. Examples of the latter category include – but are not limited to – the following: *Is_made_of, Is_located_in, Works_in, Is_workplace_of, Has_Habitat, Is_the_Inhabitant_of, Causes, Is_the_result_of, Has_color, Is_the_color_of, Is_payment_to, Is_payment_for, Wears_garment*, etc. Contrary to resources like SIMPLE (Busa et al., 2001) and the Brandeis Semantic Ontology (Pustejovsky et al., 2006), that use the *Qualia Structure* templates, our relations are concept- and domain-specific—not to mention that *Qualia Structure* is better suited to the semantic representation of nouns. The result of this encoding is a dense network of relations among entries (both single- and multi-word ones) in the lexicon.

However, mapping words to concepts already defined in the lexicon is not an easy task. This is especially true for VMWEs. Moreover, in many cases, concepts already defined for single-word entries in the lexicon are perceived of as more general or neutral and only roughly correspond to the meaning load that VMWEs bear. For example, the VMWE δαγκώνω τη λαμαρíνα in (3) denotes an emotion event, relative to the emotion love. It is mapped, therefore, onto the concept prototypically defined for the single-word entry “ερωτ ε ύ*o*μαι” (=to fall in love).

(3) δαγκώνω τη λαμαρíνα
  *lit*. bite-_1SG_ the panel_−SG.ACC_ (=to be infatuated, to have it bad)

Note, however, that the two lexical instances are not absolute synonyms in the sense that there are subtle differences in terms of the intensity of the emotion experienced. As a matter of fact, VMWEs are rarely exact synonyms of a single verbal predicate. Within this context, the major challenge faced was to account for these fuzzy cases and find out ways for capturing the semantic distance. To overcome this issue and represent differences in meaning, near synonymous entries are also linked using relations, both generic and specific for each semantic class. More precisely, the generic relation *has_troponym* links a concept that bears a more “grounded” or neutral sense with another one that signifies a shift in terms of quantity, intensity, quality, etc. For example, the verbs γ νωρíζω (=to know) and ξ έρω (=to know) are both lexicalizations of the concept [to know]; on the contrary, the VMWE παíζω στα δ άχτυλα (= γνωρíζω πoλ ύ καλ ά) is mapped onto the concept [to know well]. The two concepts are then linked *via* the troponymy relation:

(4) *has_troponym*([to know], [to know well])

Troponymy, however, is not a semantically homogenous relation (Fellbaum, 2002). In this respect, troponyms entail a shift of meaning in terms of manner, intensity, etc. The *has_troponym* relation does not reflect this difference. To remedy this shortcoming, a list of attributes (or semantic features) with either binary or scalar values have also been defined for better representing the underlying meaning. In most cases, these attributes are specific to semantic fields. For example, lexical units that belong to the semantic field emotion are assigned values for the following attributes: (a) emotion polarity, (b) emotion intensity and (c) aspect of the emotion event. In effect, these features better account for capturing the semantic distinction between near synonyms, as for example the single word verbal predicate ϕ*o*β άμαι (=to be scared), and the VMWE in (5).

(5) μoυ κóπηκαν τα ήπατα
  *lit*.me._01SG.GEN_ were-cut._03_ the livers._PL.NOM_ (=I was very frightened, I was terrified)

The VMWE is used to denote a fear emotion event that is more intense than the emotion conveyed by the single word; thus, the two predicates can hardly be encoded as being synonyms in the lexicon. Their semantic distance is captured by encoding them as related *via* the *has_troponym* relation, and the semantic distinction is highlighted by assigning the attribute *high* to the feature *Intensity*. This brings in mind the mechanism of *Lexical Functions* proposed by Mel'čuk (2006) in his Explanatory Combinatorial Dictionary in order to represent the shifts in meaning in certain types of idiomatic expressions; the only difference here is that the idiomatic expression is treated as a separate entry, with an incorporated quasi-intensifier, and not by means of one of its components taken as an functor.

### 3.3. From concepts to semantic roles: The corpus-lexicon interface

However, the conceptual representation of meaning is only one side of the coin. Semantic roles (Fillmore, 1968, 2003) have traditionally been a way to model the semantics of predicates and their arguments. The encoding of verbal predicates at this level implies the systematic mapping between syntax and semantics, basically expressed in their argument structure. After all, different perspectives to the syntax-semantics interface have shown that predicates which share the same or equivalent argument structure, with arguments that assume the same or equivalent semantic roles (or semantic features) ultimately form a homogenous semantic class and vice versa (Gross, 1975; Levin, 1993).

In our lexicon model, each verbal predicate has been assigned to a specific syntactic class based on its valency or argument structure following the Lexicon-Grammar framework (Gross, 1975). At the next step, the grammatical function of each argument and the semantic role they assume are further specified. To account for VMWEs in a similar way, we expanded the encoding of semantic roles to the arguments of the expression as a lexical unit. In this respect, we are no longer interested in the internal structure of the verbal MWE and the grammatical functions of its fixed arguments, but on the argument structure of the expression taken as a whole. In effect, the corresponding grammatical functions of the arguments of the whole expression and their semantic roles as implied by the semantics of the overall expression are identified. Therefore, in the current implementation, the *non-lexicalised* elements of the verbal MWE as opposed to the fixed or lexicalised ones are only taken into consideration and annotated as appropriate.

For example, the VMWE παíρνω χαμπ άρι (=to notice), comprises the lexicalised elements παíρνω.v (=to take) and χαμπ άρι.n (=notice). Since the semantic load of the expression is on the noun, the expression is classified as LVC. The underlying syntactic configuration of the expression is that of a verb head that is light, and its complement (direct object). This configuration is compatible with the argument structure of the verb παíρνω.v (perno, “to take”). However, the whole expression as a lexical unit assumes the meaning of a cognitive event, and as such, it is conceived of as a predicate with two arguments: the first assumes the role of the Cogniser, whereas the second has the role of the Theme of the cognitive event:

(6) [O Γιάννηϛ]_COGNISER_ π ήρε χαμπ άρι [την αλλαγ ή]_THEME_
  *lit*. The-_NOM.SG_ John_−NOM.SG_ took_−3SG_ notice_−ACC.SG_ the _ACC.SG_ change _ACC.SG_
  John realized the change

The semantic representation of the VMWE is expected to be similar to the representation of its single-word verbal counterpart καταλαβαíνω.v (=to notice or realize) as shown in (7):

(7) [O Γιάννηϛ]_COGNISER_ κατ άλαβε [την αλλαγ ή]_THEME_
  *lit*.The-_NOM.SG_ John-_NOM.SG_ noticed the-_ACC.SG_ change-_ACC.SG_
  John realized the change.

However, this is not always the case, and the argument structure of complex predicates is not realized in a uniform way. This is particularly true about VIDs. For example, the verb εξ*o*ργíζω.v (=make furious) in Greek is an Object Experiencer verb that is, a verb in which the Experiencer of the denoted emotion event is realized as a noun phrase in accusative in Object position. The cause of the event is realized as an argument, that functions as the Subject of the verb. On the contrary, in the case of the idiomatic expression (VID) ανεβ άζω τ*o* αíμα στ*o* κεϕ άλι *(*=*make furious)*, the Experiencer is realized as a nominal complement (in genitive case), whereas the cause of the emotion is realized in Subject position:

(8) [O Γιάννηϛ]_CAUSE_ μoυ_EXPERIENCER_ αν έβασε τ*o* αíμα στ*o* κεϕάλι
  *lit*.The._nom_ John._nom_ me._gen_ raised_3−sg_ the._acc_ blood._acc_ to-the head
  John made me furious.

There is no doubt that SRL is of major importance to computational systems since it provides a shallow meaning representation that is prerequisite of inferences that are not possible from the pure surface form or even from the parse tree. This is especially true for VMWEs (Fotopoulou and Giouli, 2018): lexically distinct expressions correspond to the transitive/intransitive usage depicting a single event from a reverse perspective. For example, the expressions βγ άζω απó τα ρ*o*ύχα in (9) and βγαíνω απó τα ρ*o*ύχα μ*o*υ *i*n (10) correspond to the transitive and unaccusative usage of the verb θυμώνω.v (=to make angry) depicted in (11) and (12) respectively.

(9) [O Γιάννηϛ]_CAUSE/AGENT_ έβγαλε **[**τη Mαρíα]_EXPERIENCER_ απó τα ρ*o*ύχα της
  *lit*. The._nom_ John._nom_ took-out_3−sg_ the._acc_ Maria._acc_ from the clothes hers
  John made Maria very angry)
(10) [H Mαρíα]_EXPERIENCER_ θ ύμωσε
  *lit*. The._nom_ Maria._nom_ got-angry_3−sg_

Notice that θυμώνω.v (=to make angry) is an Object-Experiencer predicate that enters the choative-inchoative alternation as shown below:

(11) [O Γιάννηϛ]_CAUSE/AGENT_ θ ύμωσε [τη Mαρíα]_EXPERIENCER_
  *lit*. The._nom_ John._nom_ made-angry_3−sg_ the._acc_ Maria._acc_
  John made Maria angry.
(12) [H Mαρíα]_EXPERIENCER_ βγ ήκε απó τα ρ*o*ύχα της
  *lit*. The._nom_ Maria._nom_ went-out_3−sg_ from the._acc_ clothes._acc_ hers._poss_
  Maria got very angry.

In this regard, our model seeks to address these issues by assigning semantic roles to the arguments of the VMWEs. The encoding of semantic roles was based on empirical data retrieved from annotation.

## 4. Empirical data: Corpus annotation

**Figure 2:**
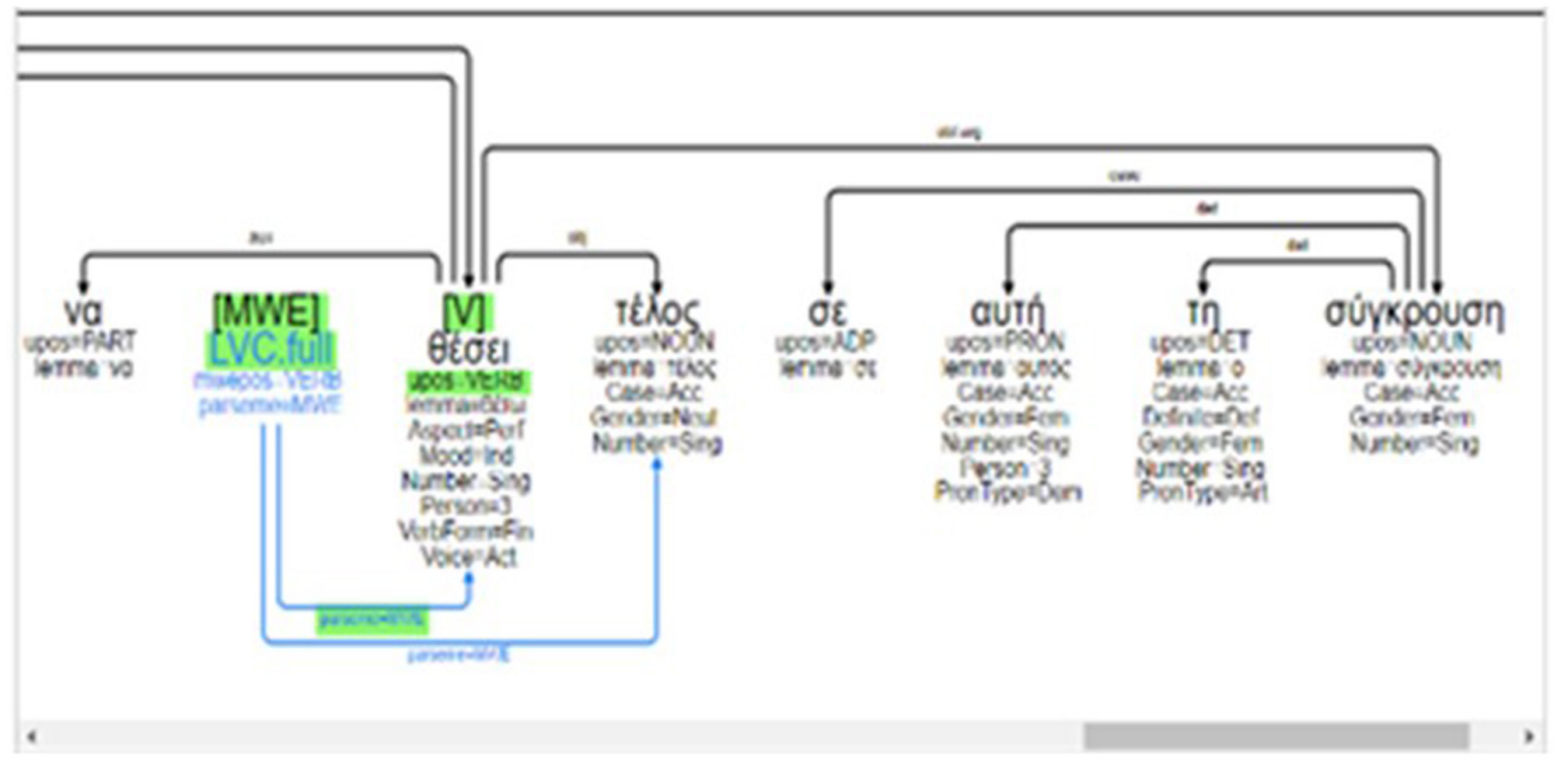
Sub-MWE annotation in GREW (Guillaume, 2021).

Annotation at the level of semantics has been applied manually on top of an existing Greek (EL) corpus that has already been annotated for VMWEs. More precisely, we used the latest version (edition 1.2) of the Greek (EL) section of the PARSEME corpus (Ramisch et al., 2020). We have chosen the PARSEME-el VMWE corpus since it is reported to have been developed following guidelines from a multilingual perspective. The EL corpus comprises textual data that originate from a variety of online sources (the Greek wikipedia, online news portals and online versions of Greek newspapers and magazines). Following the guidelines, the corpus bears manual annotations for the following types of VMWEs: VIDs, LVCs, VPCs, and MVCs. Apart from the manual annotations at the VMWE level, the corpus is coupled with lemma and morpho-syntactic information that is compatible with CoNLL-U format. Additionally, dependency parsing has been automatically performed using UDPipe (Straka and Straková, 2017) trained on UD version 2.5 (Nivre et al., 2016). An example of a VMWE annotation visualization in the dedicated GREW tool (Guillaume, 2021) is provided in Figure 2.

**Figure 3:**
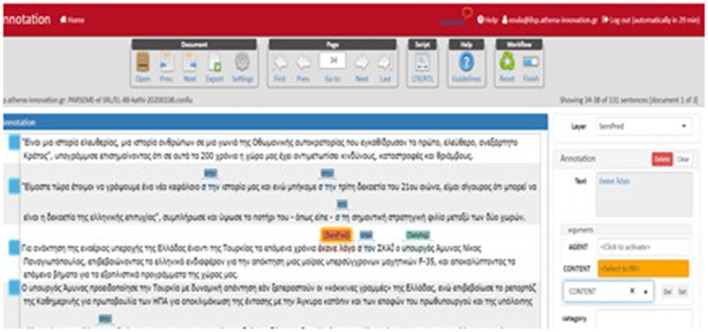
SRL annotation of VMWEs in WebAnno.

Annotation was performed manually *via* WebAnno (Eckart de Castilho et al., 2016) on LVCs, VIDs, and VPCs leaving, thus MVCs for future treatment. Prior to annotation proper, detailed guidelines were defined, and trial annotation was performed. We opted for a rather coarse set of semantic roles: Agent, Experiencer, Cogniser, Force, Theme, Result, Content, Cause, Instrument, Beneficiary, Source, and Goal, that roughly correspond to VerbNet and LIRICS proposals (Bonial et al., 2011), although VerbNet has a much larger roleset. To speed up the annotation process and to ensure consistency, detailed specifications regarding each role were elaborated and enriched with examples where applicable. Annotation was then performed as a two-step procedure. At the first stage, VMWEs that constitute semantic predicates mapped onto a concept are selected. Where applicable, a (semantically equivalent) single-word verbal predicate was used to guide the identification not only of the semantics of the VMWE, but also its arguments. For VIDs and VPCs, only verb heads were selected to overcome issues that arise from long-distance dependencies and discontinuities. In the case of LVCs, only the noun predicate was annotated. The selected markables were then annotated at the SemPred layer which is available as a WebAnno built-in module. A second span layer, namely, SemArg, represents slot fillers. The arguments of the VMWE (taken as a whole) were identified and the semantic roles they assume were further specified. This implies that the non-lexicalised elements of the VMWE as opposed to the fixed ones are mapped onto semantic roles. An example of SRL annotation of a VMWE is illustrated in Figure 3.

So far, 1,219 VMWEs (selected on the grounds of lexicographic evidence) have been encoded under the SIGNIFIED branch of our linguistic ontology and have been mapped onto concepts. However, the annotation has been performed only on a subset, that is, on c. 800 instances of VMWEs, that is, the ones that were also found in the PARSEME corpus. Of these, 379 instances were identified as VIDs, 7 as VPCs, and 425 as LVCs: in total, 811 VMWEs. Circa 10% of the VMMEs (80 VMWEs) were annotated by a second student annotator in view of calculating the inter-annotator agreement (IAA) between the two. Prior to the annotation proper, extensive discussions took place. A pilot annotation of c. 20 VMWEs identified problematic cases and discrepancies. After reaching a consensus in annotation, the second annotator worked alone annotating c. 80 VMWEs in 120 sentences. IAA was then calculated (Cohen-κ) with respect to the number of arguments identified in each sentence, and the labels assigned to them, reaching an agreement of 0.80 and 0.75 respectively. In fact, VMWEs denoting activity, emotion, cognition, movement seemed to be relatively easy to annotate and to disambiguate the semantics of their arguments. This is particularly true with VMWEs which can be mapped onto a simple event concept. In these cases, the VMWE can be paraphrased as a single-word verb predicate. LVCs seemed to be the least problematic once their sense was disambiguated. Like single-word verb predicates, issues that arise during the annotation of LVCs are relevant to the granularity of the role-set employed, or the specification of the appropriate role. In most cases, LVCs as opposed to their single-word counterparts accept only the argument denoting the Agent, Experiencer, Cogniser lacking the argument denoting Theme, etc.

As expected, SRL on VIDs was the most challenging. In fact, depending on the meaning SRL is occasionally straightforward:

(13) [o Πέρεθ]_EXPERIENCER_ έχει ϕάει χυλóπιτα
  *lit*. The_−NOM.SG_ Perez-_NOM−SG_ has_−3SG_ eaten chilopita-_ACC.SG_
  Perez has been disappointed.

Problematic cases are related to a shift in meaning and the incorporation of one or more arguments into the VMWE, diathesis alternations, or difficulties in word sense identification and/or sense mapping. For example, the VID αν*o*íγω τ*o*υς ασκ*o*ύς τ*o*υ *A*ιóλ*o*υ (=to open Aeolus bag) is semantically equivalent to the phrase “create problems”. However, only the AGENT is realized in the sentence:

(14) [O Tραμπ]_AGENT_ άν*o*ιξε τ*o*υς ασκ*o*ύς τ*o*υ *A*ιóλ*o*υ στη [Mέση Aνατoλ ή]_LOC_
  *lit*. The-_NM.SG_ Trump-_NM.SG_ opened-_3.SG_ the-_ACC.SG_ bag-_ACC.SG_ of-the-_GEN.SG_ Aeolos-_GEN.SG_ in the Mid-East
  Trump created problems in the Mid-East

Similarly, mapping the sense of VIDs like εξαπ*o*λ ύω πυρ ά (=unleash fire) to a single-word verb predicate proved to be difficult; as a result, disambiguation of their arguments proved to be problematic:

(15) [H αντιπoλíτευση]_AGENT_ εξαπ*o*λ ύει πυρά [κατ ά της κυβέρνησης]_THEME/GOAL_ [για τoυς χειρισμoύς]_CAUSE_ της
  *lit*. The opposition_−NM.SG_ unleash-_3.SG_ fire-_ACC.SG_ against the-_GEN.SG_ government-_GEN.SG_ for the_−ACC.PL_ handlings-_ACC.PL_ it's to-the issue
  The opposition accuses/attacks the government for handling the issue.

This process revealed pairs of VIDs usually with shared lexicalised elements that differ only in their fixed verb heads; these are conceived of as diathesis alternations and are encoded accordingly:

(16) [H τράπεζα]_AGENT_ βγ άζει στo σϕυρí [τo ιστoρικó ξενoδoχεío]_THEME_
  *lit*. The bank_−NOM.SG_ takes-_3.SG_ to-the-_ACC.SG_ hammer-_ACC.SG_ the-_ACC.SG_ hotel-_ACC.SG_
  The bank auctions the historic hotel
(17) [χιλιάδες σπíτια]_THEME_ θα βγ*o*υν στ*o* σϕυρí
  *lit*. thousands houses_−NOM.PL_ will go-out-_3.PL_ to-the-_ACC.SG_ hammer-_ACC.SG_
  thousands of houses will be sold at auction

## 5. Conclusion

We have presented a model for representing the semantics of VMWEs by taking into account their inherent idiosyncrasies: lexical, syntactic and semantic. The model entails a holistic approach to VMWE representation and touches upon the lexicon-corpus interface beyond providing lexical semantic relations. In contrast to dictionary models that try to model the internal structure of the MWE, our approach models argument structure taking the whole MWE as a semantic predicate. We seek to provide a shallow semantic representation for VMWEs that is similar to the semantic representation of single-word verb predicates. The model assumes the form of a linguistic ontology and has already been used to encode Greek VMWEs. The encoding of semantic properties is based on empirical data drawn from a corpus annotated at the level of semantic role labeling. Future work is underway toward enriching the lexicon with more instances of VMWEs also taking into account MWEs that belong to other grammatical categories. Moreover, inter-linking entries with other lexical resources, as for example, WordNet synsets, would be the next step. Additionally, SRL on the PARSEME corpus is still ongoing with a view to training a tool for the automatic SLR that takes VMWEs into account. Moreover, the quality of the annotation will be further ensured by obtaining more annotations to calculate inter-annotator agreement.

## Data availability statement

Publicly available datasets were analyzed in this study. This data can be found here: https://www.clarin.si/repository/xmlui/handle/11356/1555.

## Author contributions

The author confirms being the sole contributor of this work and has approved it for publication.

## Footnotes

^1^ https://parsemefr.lis-lab.fr/parseme-st-guidelines/1.2/

^2^According to Clairis and Babiniotis (2005), these adverbs have two distinct functions: as adverbs denoting time or location, they are used as modifers; combined with prepositions, they form complex prepositions, as for example μπρ*o*στ ά απó (=in front of), μέσα σε (=in), π άνω απó (=over), etc.
